# Hypertrophy and Dilatation of the Heart, with Hydro-Pericardium

**Published:** 1859-01

**Authors:** B. Woodward

**Affiliations:** Galesburg, Ill.


ARTICLE III.

### HYPERTROPHY AND DILATATION OF THE HEART, WITH HYDRO-PERICARDIUM.

BY B. WOODWARD, M.D., GALESBURG, ILL.

Was called, June 30th, 1858, to see Mr. Wichstrom, a Swede, aged thirty-two. Has been out of health for more than a year, and since last December has not been able to work at his trade (shoemaker) much of the time; has been confined to his bed a week, but has had no treatment. Has suffered from palpitation of the heart for many years; was sick five years ago in Sweden; does not know what the doctors called his sickness, but had pain in his side and cough. Has had pain in his joints (rheumatism?) at various times, so that he could not work for weeks at a time. I found him with clean moist tongue; no heat of surface, but perspiring profusely; has no cough or expectoration, and no pain anywhere, but "cannot breathe;" pulse 160 per minute; respiration 55 per minute; character of pulse hard and jerking, showing labored action of the heart; respiration short, and the lungs do not inflate well, particularly the left; in breathing, the abdominal muscles are brought into active exercise; his face and lips have a peculiar livid and motled appearance; mind perfectly clear; percussion gives a clear resonance over the right upper part of thorax; on the left side, extending from the second rib to the lower part of the thorax, there was a perfectly dead sound like solid flesh; the heart's action was so violent that its impulse could be plainly seen at the distance of ten feet, and communicated to the muscles of the abdomen a distinct tremor; the sounds of the heart were quite audible two feet from the bed. The only position in which he could lie was on his back, with his head low. If he turned on his side there was a sense of suffocation, and if he sat up vomiting was induced. Either with or without the stethoscope it was impossible to make out the peculiar sounds of the heart, for it was nothing but a confused, tumultuous sound all over the præcordial region. I tried to find the bellows or blowing sound, but could not make it out. (I suspected valvular disease.) There was one peculiarity in the sounds, and that was a flapping as though there were a loose valve flapping back and forth in the right ventricle. There was great enlargement of the præcordial region, giving the cartilages of the ribs a perceptible curve.

My diagnosis was hypertrophy of the heart and effusion of serum in the pericardium, and I thought also pleuritic adhesions on the left side.

As he and his friends wished to know what I thought of the case, I told them he could live but a short time. The only treatment I tried to institute was to control the heart's action, but in vain; nothing I could do seemed to have any effect. I could find no other evidence of disease than what I at first diagnosed. The rapidity of the heart's action and the difficulty of breathing increased until the morning of the 3d of July, when he died.

Our county society being in session on the 3d, I gave a history of the case and my diagnosis of it, and invited the members present to attend a post-mortem examination the next day.

*July 4th, 8 a.m.*—Made a post-mortem examination. Present, Drs. Taylor, Spaulding and McCady. The weather being extremely warm, and the body having been put in a coffin and fastened down, it was so very offensive that we carried the examination no further than the thorax. Turning back the flaps and sawing through the ribs three inches from their sternal attachments, we found the heart occupying the whole of the left side of the anterior regions, and three inches beyond the sternum on the right side. The pericardium contained thirty-two oz. of bloody serum. The heart, when divested of everything but its own substance and emptied of blood, weighed thirty-four oz. (two lbs. two oz.) The left lung was crowded to the back and compressed to less than one-half its normal size, completely hardened like firm liver, and closely adherent to the walls of the thorax—so much so that it had to be dissected off. The right lung was healthy but engorged.

*Examination of the heart at my office.*—Weight, 34 ounces; fatty degeneration to some extent; aorta enlarged; semi-lunar valves thickened and slightly ossified; left auricle enlarged and thickened; the valve normal; left ventricle enlarged and thickened; the walls $1\frac{7}{8}$ inches thick; ascending venæ cava enlarged; right auricle enlarged; valve normal; right ventricle enlarged, with a fibrinous mass 2 lines thick and $1\frac{1}{2}$ inches long by $\frac{3}{4}$ inch broad, having slight attachments to the apex of the ventricle floating in its cavity. There were small fibrinous deposits adherent to the walls of the right auricle; these deposits looked and felt like plates of fibrin which had been washed out. The left ventricle would have contained a large goose egg. The notes of the dissection of the heart and its admeasurements were kindly taken by my friend, J. W. Spaulding, M.D.

There are several points of interest in the above case. Hope, Joy, and others, speak of hydro-pericardium without the co-existence of serous effusion into the cavity of the pleura or a general dropsical condition, as so rare an affection as to cast a doubt over those cases in which it has been said to exist. In the present case there can be no doubt on the subject, as the thorax was opened with great care, having this very point in view. *There was no serous effusion in the cavity of the chest*, and when the pericardium was opened it was done with a small puncture, and the serum carefully absorbed with a sponge and wrung out into a basin.

The position chosen by the patient is another point of interest. He could not sit up or lie on either side. In Dunglisson's *American Intelligencer* for 1842, p. 22, will be found an article from the pen of Dr. John Mackenzie, in which he states that "the recumbent position is often chosen by patients laboring under hydrops-pericardi, in order to relieve the diaphragm from the pressure which would otherwise be thrown upon it; while in hydro-thorax they *cannot* take this posture, for the water would be thrown back so as to press on the roots of the lungs and prevent the ingress of fresh air; that in hydro-pericardium this last condition cannot take place, the water being enclosed in a shut sack."

From the very imperfect history of the case which I was enabled to get, I was led to the belief that the disease of the heart was originally caused by rheumatism, and the extensive adhesions of the left lung had been caused either by pneumonia or pleurisy. The existence of the fibrinous flap (for I know not what else to call it) in the right ventricle I cannot account for—I suppose it to be a species of polypus. The immense size and weight of the heart is a very curious circumstance. I have made a wet preparation of the heart and preserved it.

Perhaps it may be thought presumptuous in me to present the history of this case and the autopsy so soon after the case of Pus Blanchard's child. It is not from any mania for writing, but it seems to me that we have not autopsies enough. It is by post-mortem examinations that we either verify or disprove our diagnosis of cases. In either event we have pathology, and all correct practice must be based on pathology. By this we separate ourselves most effectually from quacks of all kinds—they never seek to have their cases submitted to the rigid test of morbid anatomy.

